# Vascular Involvements in Cholangiocarcinoma: Tips and Tricks

**DOI:** 10.3390/cancers13153735

**Published:** 2021-07-25

**Authors:** Roberta Angelico, Bruno Sensi, Alessandro Parente, Leandro Siragusa, Carlo Gazia, Giuseppe Tisone, Tommaso Maria Manzia

**Affiliations:** 1 Hepatobiliary Surgery and Transplant Unit, Department of Surgical Sciences, University of Rome Tor Vergata, 00133 Rome, Italy; roberta.angelico@gmail.com (R.A.); bruno.sensi@ptvonline.it (B.S.); aleparen@gmail.com (A.P.); leandro.siragusa@alumni.uniroma2.eu (L.S.); carlo.gazia@ptvonline.com (C.G.); manzia@med.uniroma2.it (T.M.M.); 2 Department of Hepatobiliary and Pancreatic Surgery, Queen Elizabeth Hospital, Birmingham B15 2TH, UK

**Keywords:** cholangiocarcinoma, vascular involvement, resection, vascular reconstruction, liver, outcomes

## Abstract

**Simple Summary:**

Cholangiocarcinoma (CCA) is the second most common liver primary malignancy and its gold-standard treatment is surgery. Unfortunately, CCA is seldom amenable to curative resection due to late-stage diagnosis and frequent major vascular invasion. Major vascular invasion has historically been considered a contraindication to resection, but lately aggressive surgeries for CCA with vascular involvement have been shown to improve outcomes. The purpose of this review is to provide a comprehensive and up to date summary of the strategies for CCA resection, focusing on the surgical techniques and results of complex procedures with tumour vascular involvements. The current review shows that satisfactory results can be achieved in patients with CCA and tumoral vascular invasion by aggressive surgical resection and challenging vascular reconstruction, ensuring a meticulous evaluation of patients in a multidisciplinary setting by experienced hepatobiliary surgeons.

**Abstract:**

Cholangiocarcinoma (CCA) is an aggressive malignancy of the biliary tract. To date, surgical treatment remains the only hope for definitive cure of CCA patients. Involvement of major vascular structures was traditionally considered a contraindication for resection. Nowadays, selected cases of CCA with vascular involvement can be successfully approached. Intrahepatic CCA often involves the major hepatic veins or the inferior vena cava and might necessitate complete vascular exclusion, in situ hypothermic perfusion, ex situ surgery and reconstruction with autologous, heterologous or synthetic grafts. Hilar CCA more frequently involves the portal vein and hepatic artery. Resection and reconstruction of the portal vein is now considered a relatively safe and beneficial technique, and it is accepted as a standard option either with direct anastomosis or jump grafts. However, hepatic artery resection remains controversial; despite accumulating positive reports, the procedure remains technically challenging with increased rates of morbidity. When arterial reconstruction is not possible, arterio-portal shunting may offer salvage, while sometimes an efficient collateral system could bypass the need for arterial reconstructions. Keys to achieve success are represented by accurate selection of patients in high-volume referral centres, adequate technical skills and eclectic knowledge of the various possibilities for vascular reconstruction.

## 1. Introduction

Cholangiocarcinoma (CCA) is a rare cancer, yet the second most common primary liver cancer after hepatocellular carcinoma. The incidence of CCA has been increasing over the past decades, affecting 0.5–2.0 in 100,000 individuals in Western countries and approximately 100 in 100,000 individuals in Thailand, and is becoming a global health problem [1]. CCA arises from the biliary tree and is divided into three subgroups, based on its localisation: intrahepatic CCA (iCCA), hilar/perihilar CCA (pCCA) and distal CCA (dCCA). The anatomical distinction between pCCA and dCCA is represented by cystic duct insertion, whereas iCCA emerges from the secondary order intrahepatic bile ducts. Up to 80–90% of CCAs are extrahepatic (pCCA, dCCA), while the remaining 10–20% of lesions are iCCA [2].

Surgical treatment is the gold standard for CCA, but unfortunately, the tumour is frequently diagnosed in late stages due to its asymptomatic course, resulting in unresectable disease at diagnosis. In fact, CCA frequently involves major hepatic vessels, such as the inferior vena cava (IVC) and/or hepatic veins, portal vein (PV) and hepatic artery, which might limit the surgical strategies.

In the past decades, many efforts have been made by the surgical community worldwide to push the boundaries on surgical management of CCA [3]. The surgical indications for CCA have been constantly expanded, and aggressive approaches to CCA involving vascular structures have been pursued with satisfactory outcomes. While iCCA and pCCA share similar surgical approaches involving liver resection, dCCA requires duodeno-pancreatic surgery. Therefore, for the sake of clarity and concision the current review is limited to iCCA and pCCA, and does not cover dCCA.

The aim of this manuscript is to review the main surgical techniques described for iCCA and pCCA with vascular involvement, with a particular focus on vascular resection and reconstruction.

## 2. Materials and Methods

A comprehensive review using the PubMed database with the goal to investigate the current management of vascular involvement in CCA was performed. Data were retrieved from published registries, case series and trials reporting surgical outcomes after vascular resection and reconstruction, combined with liver resection, during operations for CCA. Inclusion criteria for this review were as follows: (1) any study (original article, systematic review, case reports, case series, trials) including liver resection for intrahepatic or hilar/perihilar CCA with vascular involvement; (2) studies in which resection and/or reconstruction of PV, hepatic artery, IVC and/or hepatic veins for hilar or intrahepatic CCA were reported; and (3) peer-reviewed manuscripts written in English. The references of each of the selected articles were also evaluated in order to locate additional studies that were not included during the initial search. Reports that included or were mixed with distal CCA were excluded.

The relevant articles were extracted independently by three authors (A.P., B.S., C.G.) who evaluated and excluded duplicates. No specific search dates were used. Consensus for the relevance of an included study were carried out by two senior authors (R.A., T.M.M.) who are experts in the field. Given the heterogeneity of the selected studies and paucity of patients identified within the selection criteria, the results are reported as a narrative review.

## 3. Intrahepatic Cholangiocarcinoma with Vascular Involvement

### 3.1. Oncological Considerations

Vascular involvement in the setting of iCCA can be distinguished into three different subsets: (1) microscopic/microvascular invasion; (2) macroscopic invasion of a major vessel ipsilateral to the lesion; and (3) macroscopic invasion of a major vessel of the contralateral liver, namely the future liver remnant (FLR).

Microvascular invasion is diagnosed histologically after surgical specimen excision and is not predictable preoperatively. The presence of iCCA with microvascular invasion represents a negative prognostic factor; in a large, multivariate analysis study of 1333 patients diagnosed with iCCA, the authors found that vascular invasion was an independent predictor of poor survival (*p* = 0.011) [4]. The one-, three- and five-year overall survival (OS) rates in patients with or without vascular invasion were 56.8%, 16.5% and 9.4%, and 58.5%, 26.8% and 18.5%, respectively (*p* = 0.002). Of note, no significant difference in survival was found between patients with macroscopic (*n* = 106) and microscopic (*n* = 100) vascular invasion (*p* = 0.790) [4]. In addition, the same authors analysed the survival rates according to the surgical margin status, demonstrating that when complete tumour resection (R0) is achieved, results are markedly improved. In fact, one-, three- and five-year OS rates in R0 and R1 were 79.1%, 42.6% and 28.7%, and 60.5%, 20.1% and 13.9%, respectively, and fell to 0% at five years after surgery for patients with R2 resection.

Since this challenging surgery is the only option for these patients, the current guidelines of the American Hepato-Pancreato-Biliary Association (AHPBA) and the European Network for the Study of CCA recommend surgery for patients with preoperatively diagnosed macrovascular invasion [2,3].

Macrovascular invasion of a major vessel of the theoretical FLR has historically been considered a contraindication to resection. In the past, some of these patients were considered candidates for liver transplantation (LT), but the initially reported poor outcomes have established iCCA as a contraindication for transplantation. Nowadays, with the expansion of the concept of “transplant oncology”, there has been renewed interest in LT as treatment for CCA, but vascular invasion is still considered an exclusion criterion. Two trials are currently ongoing to investigate whether transplantation might be an option in select patients with local advanced iCCA that has been stable for at least six months after neoadjuvant therapy (ClinicalTrials.gov Identifier: NCT04556214 and ClinicalTrials.gov Identifier: NCT04195503) [5,6]. Nonetheless, the International Liver Transplant Society consensus defined that LT for CCA should be performed only under strict clinical trials, remaining an option only in the “experimental” setting [7].

Other options have been developed throughout the years to extend the possibility of surgical resection for these patients. In general, these strategies aim at complete resection of the tumorous mass through vascular resection of the affected vessel, followed by reconstruction to preserve the liver remnant.

iCCA may involve any major vessel in the liver, including “posterior/central” pattern when the tumour extends to the main hepatic veins or IVC, and “hilar” pattern when it invades the portal structures. The latter, however, is more common with pCCA. Major vascular resections can be used successfully to obtain R0 resection in up to 84% of patients [8]. In the largest study to date, morbidity (41.9% vs. 55.5%; odds ratio (OR) = 0.68, 95% confidence interval (CI) = 0.32–1.45) and mortality (7% vs. 8.2%; OR = 1.05, 95% CI = 0.32–3.47) rates have been shown to be comparable between patients undergoing vascular resection (*n* = 128; 21 IVC resections, 98 PV resection, 9 combined vascular resections) and those treated by standard resection (*n* = 1087) [9]. In the same study, the median recurrence-free rates and the OS rates were similar between the groups. Another recent study comparing hepatectomies with vascular resection, major hepatectomies and the combination of the two, did not find any differences in terms of postoperative complications among the three groups [10]. Based on these data, current guidelines consider resectability for iCCA as a possibility to completely remove the disease with curative intent (parenchymal free tumour margin, R0) while ensuring an adequate FLR, even when the tumour invades the portal or hepatic veins/IVC (V status) [2,3]. Furthermore, in recent years, some authors have pushed the boundaries of liver surgery with the first reports of major two-staged resection combined with ex situ liver surgery [11,12].

When iCCA presents with vascular invasion, any of the major involvement patterns has its own significance and treatment. Among these, IVC involvement represents a major surgical challenge. In the literature, iCCA represents an indication for liver and IVC resection in 20–33% of cases [13,14]. In the last few decades, many studies have demonstrated the feasibility of hepatectomies combined with IVC resection, obtaining R0/V1 resection in most patients [15]. However, morbidity and mortality rates are still high when IVC resection is performed, ranging from 42% to 64% and 4.3% to 19.5%, respectively [13,16,17,18]. Post-hepatectomy liver failure (PHLF) and sepsis represent the main causes of death, while complications directly related to IVC resection and reconstruction (e.g., graft infection or thrombosis, lower limb oedema, deep vein thrombosis, pulmonary embolism) were reported in approximately 2.5–5% of cases in a recent review [14]. In particular, the incidence of graft thrombosis after IVC resection/reconstruction is 2.5% [14]. Given the relatively limited data thus far reported, the long-term outcomes of patients undergoing combined liver and IVC resection for iCCA have been reported in only a few studies and range from 11.6% to 37% at five years of follow-up [14]. Although prognosis remains poor, these outcomes should probably be considered acceptable, given the absence of alternative options for cure.

Hepatic vein infiltration also necessitates resection followed by reconstruction, and outcomes result from a very small series with postoperative mortality of approximately 12%, which is comparable to outcomes obtained after IVC resection [19].

In addition, PV resection for iCCA has been specifically investigated in a few studies [9,20]. Reames et al. reported the largest experience of PV resections (*n* = 98) and showed no difference compared to standard hepatic surgery [9].

Lastly, hepatic artery resection for iCCA is relatively rarely undertaken and there is a scarcity of literature on this topic.

When this extreme liver surgery is not feasible, neoadjuvant therapy (NAT) should be considered for two reasons: firstly, NAT can convert as many as 53% of patients with previously unresectable disease to secondary resectable disease [21], and secondly, NAT may serve as a selection process, with progression of patients unlikely to benefit from surgery and those with stable or regressing lesions who are viable candidates for extreme resection [22].

### 3.2. Surgical Approaches in iCCA with Vascular Involvement

#### 3.2.1. Inferior Vena Cava Involvement

Site and depth of IVC involvement are fundamental for operative planning, as different strategies have been developed for increasingly complex situations. The various approaches are described consequentially, beginning with the “simpler” cases to the most challenging ones, requiring “very complex” resection attempts. Table 1 summarises the main studies on the subject.

##### Minor IVC Involvement below the Hepatocaval Confluence 

**Figure 1:**
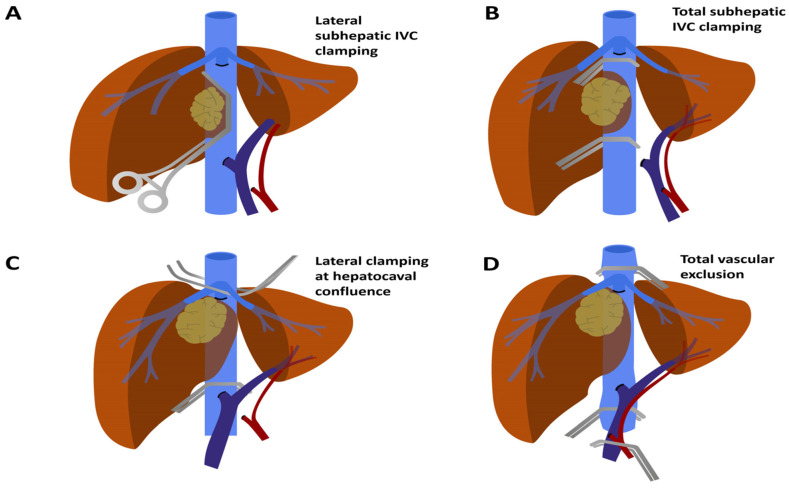
Different IVC clamping strategies depending on infiltration site. (**A**) Lateral IVC clamping below hepatocaval confluence: this strategy can be readily advantageous when involvement is <60–120° circumferentially and <2–3 cm longitudinally. (**B**) Total IVC clamping below hepatocaval confluence: this permits venous return from the liver and does not require hepatic ischaemia (**C**) Side clamping at hepatocaval confluence is a strategy to permit venous return from one side of the liver, despite “very high” neoplastic infiltration. (**D**) Total vascular exclusion necessitates complete IVC clamping above the hepatocaval confluence, concurrent Pringle manoeuvre and hepatic ischemia. Abbreviations: IVC, inferior vena cava.

Resection of the cancer could necessitate resection of the IVC itself to ensure radicality. When the IVC is involved below the hepatocaval confluence, it is theoretically possible to clamp it by different techniques (Figure 1) without affecting drainage from the liver/FLR, thus minimising hepatic ischaemia time.

If IVC involvement is tangential/minor (<60–120° of its circumference and <2–3 cm longitudinally) [24,27], it may be possible to side clamp the vein without complete interruption of flow through the vessel (Figure 1A). This situation is highly advantageous as it minimises derangement in physiologic functions, avoiding pre-load reduction, splanchnic stasis and ischaemia to vital organs.

Complete IVC isolation and liver mobilisation is mandatory at the early step of the operation. In only exceptionally large cancers when rotation of the liver from the IVC might be not feasible, a primary anterior approach could be justified and transection of the liver parenchyma may initially be performed to expose the retro-hepatic IVC [23,28]. With the exposed IVC as the only remaining tumour site, a side-biting clamp could be placed, and thus, it is possible to resect the cancer and anterior IVC wall, completing the hepatectomy. The use of a Pringle manoeuvre to minimise bleeding is legitimate, but not mandatory; however, the ideal balance between parenchymal ischaemia and blood loss may be often difficult to achieve [29].

**Figure 2:**
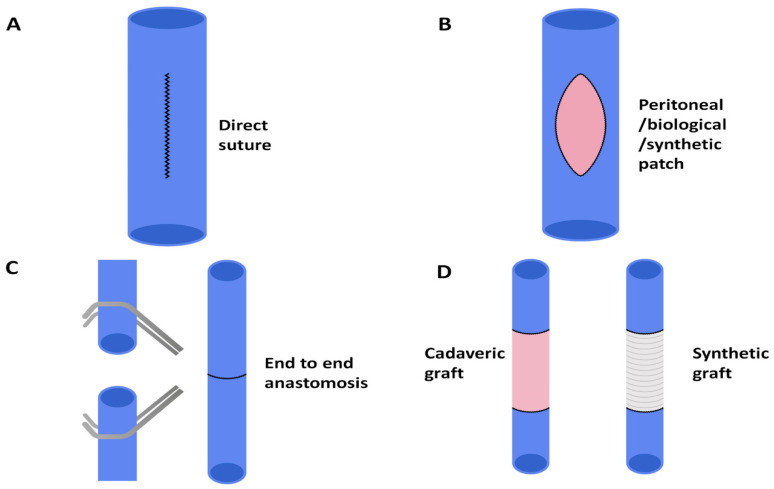
Reconstructive options after IVC resection. (**A**) Direct suture is possible when resection involved less than 120° of the IVC wall. Transverse repair (not depicted) may be used to lower chances of stenosis for long longitudinal defects. (**B**) Patch repair can be used when less than 180° of the IVC has been resected. Peritoneal, biological and synthetic options exist. (**C**) End-to-end anastomosis can be fashioned for circumferential IVC resection <3 cm in length. (**D**) Interpositional grafts are the preferred option for defects longer than 3 cm and autologous, cadaveric and synthetic grafts have been used.

In these cases, vascular reconstruction can be straightforward: if resection affects less than 120° of the IVC wall, it is possible to proceed with direct suture repair without risking excessive luminal narrowing (Figure 2A). Direct repair may be longitudinal in small or transverse defects to minimise risk of stenosis [27,29]. In this regard, a recent review investigating the safety and efficacy of IVC reconstruction during liver resection demonstrated that in cases in which tangential resection of the cava was required without IVC replacement (*n* = 118), primary closure of the IVC defect was possible with direct suturing in up to 86 (72.9%) of the cases, whereas only 32 (27.1%) patients required the use of a polytetrafluethylene (PTFE) graft [14].

##### Major IVC Involvement below Hepatocaval Confluence

When IVC involvement is greater than 60–120° circumferentially and/or 2–3 cm longitudinally, side clamping could be impossible and a full IVC clamp might be mandatory. In this scenario, it is appropriate to place clamps above the renal veins and below hepatic veins whenever possible in order to minimise liver ischaemia and splanchnic stasis (Figure 1B).

Although collateral circulation would partly replace IVC flow, porto-systemic shunting should be taken into consideration.

Parenchymal resection is often carried out using an anterior approach, as described in the previous paragraph. Alternatively, some authors have proposed a different technique: after dissection, ligation and division of inflow and outflow vessels of the affected hemi-liver, the IVC is clamped and resected, and after IVC reconstruction and re-established flow, the parenchymal transection might be undertaken in a very controlled setting [30].

In any case, the vascular reconstructive phase is more complex. If less than 180° of the IVC has been resected, patch repair can be fashioned (Figure 2B). In a recent systematic review, patch repair was required in up to 13% of IVC resections [18]. Patch vascular repairs might be performed using: (1) autologous materials, such as the peritoneum or external iliac vein; (2) biological materials, such as bovine pericardium; and (3) synthetic grafts, including Dacron^®^ (polyethylene terephthalate) and Gore-Tex^®^ (PTFE).

A peritoneal patch can be retrieved from the peritoneum anterior to the renal fascia, right hypochondrium, falciform ligament or the anterior abdominal wall (possibly including the posterior rectus sheath), trimmed to fit the defect and sutured in place [31,32,33]. Pulitano et al. used a peritoneal patch for 21 IVC repairs, reporting no vascular complications related to the patch [31]. The advantages of this technique include very low risk of thrombosis, greater resistance to infections and absence of costs. Sano et al. published their results using an autologous external iliac vein patch, in which they report no thrombosis nor transient oedema of the lower limb as complications [34]. Autologous vein grafts are optimal patches, yet they often require an additional incision, which may lead to complications and increased operative time. Bovine pericardial patches have also been used with good results, although the cost of this modality is high [35].

In many studies, surgeons chose synthetic patches, mainly Dacron^®^ and Gore-Tex^®^ [13,20,21]. The disadvantages of synthetic grafts include higher rates of thrombosis and infection, rigidity and cost. Nonetheless, in the largest studies to date, thrombotic events seem very rare and outcomes are acceptable [23,36]. When a synthetic patch is used, anticoagulation drugs are commonly adopted, but regimens used are highly variable. In-hospital anticoagulation with low-molecular-weight heparin (LMWH) or heparin infusion seems to be the most common peri-operative management [11,24,32,37]. Long-term anticoagulation with LMWH or warfarin and the treatment duration to prevent graft thrombosis are still controversial [14]. Potential benefits may exist for patients with previous venous thromboembolism, coagulopathy due to massive intraoperative bleeding and transfusion, large tumours or undergoing re-implantation of hepatic or renal veins [14]. Currently, there is no evidence to recommend one material for repair over another.

When more than 180° of the vascular wall needs to be sacrificed, complete resection of the infiltrated segment of IVC is required, followed by IVC reconstruction, which might be performed by different techniques. The first reconstructive option is the primary anastomosis, which has been described for small resections <3 cm in length [23] (Figure 2C). The advantages of reconstruction by primary anastomosis include the rapidity of performing a single anastomosis, no further dissection in other districts (required for autologous graft), no antigenicity (occurring with cadaveric graft) and avoiding the use of synthetic grafts, which increases thrombotic and infectious risks, as well as cost effectiveness. However, primary anastomosis might be associated with the possible disadvantage of developing tension on the anastomosis, with potential narrowing.

When the direct primary anastomosis of IVC is not suitable, an interpositional graft might be required (Figure 2D). Synthetic grafts have been extensively used and demonstrated to be very reliable [13,23]. Alternatively, the resected IVC may be substituted by autologous conduits obtained by the iliac vein or peritoneum; however, although generally safe, a few reports of thrombosis with these grafts (probably due to the calibre difference between IVC and graft) have emerged in the literature [38,39]. Papamichail et al. described IVC reconstruction using cadaveric IVC graft without post-operative vascular complications [18]. For other hepatic tumours invading the IVC, aortic grafts from deceased donors were also adopted, with the possible advantage of reducing the risk of vessel collapse due to its thickness [40]. Moreover, cadaveric grafts have the possible advantage of not requiring long-term anticoagulation therapy [41].

In some cases, although the tumour does not directly involve the hepatocaval confluence, it might be necessary to clamp the IVC above the hepatic veins. This is achieved through successive orderly clamping of the infra-hepatic IVC, hepatic hilum and supra-hepatic IVC, obtaining total vascular exclusion (TVE) (Figure 1D). Given the significant influence on cardiac pre-load, in some cases, it might be necessary to perform a portocaval shunt (PCS) in order to maintain haemodynamic stability. In this setting, haemodynamic tolerance to TVE has been defined as a decrease in mean arterial pressure of at least 30% or a decrease in cardiac index >50% [17]. When TVE is predicted to be in place for long periods of time, systematic veno-venous bypass set-up should be strongly recommended (the fashioning of veno-venous bypass is described in the following paragraph). A threshold of 60 min of TVE is cited in most studies as being an indication for veno-venous bypass, reducing blood loss and respiratory complications [42]. One study on TVE found maximum diameter of the lesion, preoperative PV embolisation and planned vascular reconstruction as independent predictors for TVE > 60 min [37]. Physiological circulation is restored by de-clamping the supra-hepatic cava, followed by the infra-hepatic cava, and finally, the PV and hepatic artery.

In a systematic analysis of liver and vena cava resection with or without TVE, without the use of perfusion strategies, operative mortality was 8% (nine of 111 cases) and graft patency was 98.2%. The liver can tolerate TVE for a limited amount of time and when complex surgery lasting more than 60–90 min is predicted, an effort to minimise ischaemic damage should be sought [43].

A “trick” to reduce TVE time and liver ischaemia when reconstructing the IVC is to “slide down” clamps below the hepatocaval confluence as soon as possible (for instance, right after the supra-hepatic anastomosis is fashioned, before construction of the inferior anastomosis [17,30,44]). Other authors have described the possibility of switching from TVE to oblique clamping of the IVC after transection of the parenchyma if the reconstruction involves only part of the vena cava and can be performed with a patch, obtaining good results in minimising TVE time [45] (Figure 1C).

**Figure 3:**
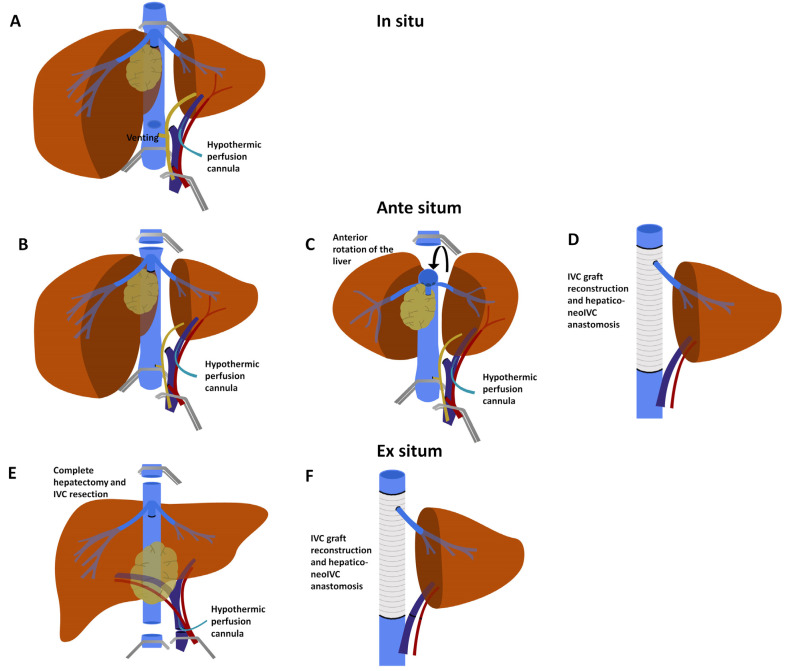
Hypothermic perfusion strategies. (**A**) In situ hypothermic perfusion: total vascular exclusion is in place and a perfusion cannula is inserted in the portal vein. A cavotomy is also performed, to be used for venting. (**B**) Ante situm technique: total vascular exclusion is in place, hypothermic perfusion is used and the vena cava has been sectioned below the superior clamp. (**C**) The liver can now be rotated anteriorly towards the surgical team, to perfectly expose the caval plane. (**D**) Resection is completed and reconstruction requires grafting of the IVC and hepatic vein re-implantation. (**E**) Ex situ technique: TVE and hypothermic perfusion are followed by PV, hepatic artery and biliary division and subsequent complete hepatectomy with IVC resection. (**F**) Reconstruction requires IVC grafting and portal and arterial anastomosis. Biliary anastomosis is also required (not depicted).

To minimise liver ischaemic damage during TVE, in 1974, Fortner et al. described in situ hypothermic perfusion (HP) [46] (Figure 3A).

This technique warrants TVE, followed by in situ perfusion of the liver with a hypothermic cytoprotective solution combined with cooling of the organ surface. Ringer’s lactate solution, chilled to 4 °C and with 5 mg/L heparin, was used. HP is delivered through cannulae inserted in the portal system distal to the occluding clamp. Cannulation can occur either through the main PV, or more conveniently, through the contralateral PV, which will be resected with the specimen. The original description also included arterial HP through the gastroduodenal artery, although this is not considered strictly necessary today. The perfusate is then drained through a cavotomy, which is usually placed just superior to the inferior caval clamp. In situ HP permits longer ischaemia times and gives the opportunity for a meticulous and careful approach to the difficult resection. Before re-perfusion, flushing at room temperature with a potassium-poor solution is recommended. The HP procedure seems to be effective in increasing hepatic tolerance to ischaemia. In one study, TVE with HP resulted in significantly lower liver function test peaks during the postoperative period when compared to TVE without HP, and especially when TVE without HP was prolonged for more than 60 min [37]. In this same report, there were no differences in mortality, although the cohort might have been too small to highlight any effect (20 vs. 49 patients). Nonetheless, mortality and morbidity of this extreme surgery remain high (Table 2). In the largest series to date, Azoulay et al. describe 19.5% mortality and 27.3% major morbidity [13]. The predictors for 90-day mortality were Charlson Comorbidity Index > 3 [47], tumour size >10 cm and the 50/50 criteria on postoperative day 5 (which included prothrombin time <50% and serum bilirubin > 50 µmol/L or 2.92 mg/dL) [48]. The one-, three- and five-year OS rates were 67%, 42% and 11.6%, respectively [13].

##### IVC Involvement at Hepatocaval Confluence

This complex situation demands TVE and challenging hepatocaval reconstruction might be required. For this reason, the use of HP is often contemplated and veno-venous bypass should be considered. Furthermore, to obtain sufficient IVC length for clamping, resection and anastomosis, the surgeon may need to use the intrathoracic portion of the vein. The latter can be accessed either by an intraabdominal, trans-diaphragmatic approach or by median sternotomy.

A veno-venous bypass should be planned preoperatively, entailing percutaneous femoral and jugular catheter placement and intraoperative cannulation of the inferior mesenteric vein (which is ligated at procedure’s end). Some groups have proposed the immediate reconstruction of the IVC with a temporary PCS and without bypass [49]. Other authors used this technique without PCS, while others did not reconstruct the IVC immediately or used bypass; only a temporary PCS was utilised [50,51].

In these circumstances, HP and complex vascular resection/reconstruction can be performed in three ways: in situ, ante situm (ex situ in vivo) or ex vivo. The in situ approach is convenient when the hepatic vein of the FLR does not require resection and re-implantation in the neo-cava; the other two techniques (ante situm and ex vivo) possess specific advantages when all hepatic veins are involved, along with the IVC.

The ante situm operation was first described in 1991 by Hannoun [52] (Figure 3B–D). This technique entails complete mobilisation of the liver and vena cava, TVE and HP through the PV, followed by sections of the hepatic veins or the vena cava itself [40]. This allows complete rotation of the liver (ex situ), giving full access to the posterior liver, cava and hepatocaval confluence, while preserving the hepatic pedicle (in vivo) and reducing the risk of vascular complication into the hilum vessels, especially to the hepatic artery. In this way, the surgeon gains operative access to a region otherwise particularly difficult to approach, representing a critical step in the operation, especially when complex resection and reconstruction are required. The ante situm technique seems to be a valid option, with mortality ranging from 0% to 14%, depending on the few available series [16,52,53,54]. In 2018, Ye et al. reported their extensive series with this technique, reporting only one death (4.3%) out of 23 patients, stressing how poor outcomes can be minimised with experience [16].

The ex vivo approach, described by Pichlmayr in 1990, requires all the steps used in the ante situm approach, followed by careful section of the hepatic pedicle and excision of the liver from the patient’s body [55] (Figure 3E,F). Resection is then carried out on the “back table”, permitting complex vascular resection and reconstruction in a most convenient setting. Once ex vivo resection is completed, autotransplantation of the remnant liver is required, generally performing vascular anastomoses in the same order as for LT (suprahepatic cava, infrahepatic cava, portal, arterial and biliary). The main advantage of the ex vivo procedure is maximal control over resection and reconstruction. The disadvantages include the need to re-implant, making the procedure a true autotransplantation. However, in cases in which both hilar and outflow structures are compromised, this might be the only surgical option.

The results of pioneering studies, even from highly experienced centres, have shown a prohibitively high mortality rate of 32% [49]. Nonetheless, in a recent meta-analysis of 244 patients treated with ex vivo surgery, complete tumour resection was achieved in 98% and 30-day mortality was 8%, not far from the results of “ordinary” liver resection [56]. However, due to the possible complications of this complex procedure (i.e., thrombosis, pulmonary embolism, renal impairment), the 90-day mortality would probably better reflect the outcomes of this type of surgery, rather than the 30-day timepoint.

Despite this, there is no evidence to support one HP technique over another, and the choice should be selected according to the tumour size and position, vascular involvement and the surgeon’s skills. Shen et al. reported the only study comparing the in vivo and ex vivo techniques in 71 patients with end-stage hepatic alveolar echinococcosis involving the IVC [27]. In their series, 26 patients in the in vivo group and 45 patients in the ex vivo group had similar postoperative morbidity (26.9% vs. 24.4%) and mortality (11.5% vs. 6.7%). In this retrospective study, patients undergoing ex vivo procedures had larger lesions, most frequently involving the portal structures, and underwent lengthier procedures with longer periods of ischemia, requiring more blood products. Overall results were very promising, yet this group of patients suffered from a benign disease that permitted conservation of large percentages of liver parenchyma, featuring low rates of postoperative liver failure.

Only a few cases of vena cava resection without reconstruction have been reported, possibly due to the extensive collateral circulation that forms after complete obstruction of the IVC by the mass [27].

#### 3.2.2. Hepatic Venous Involvement

When iCCA invades the contralateral hepatic vein, its resection and reconstruction is the only option to achieve complete tumour excision. The surgical approach includes complete mobilisation of the liver from the vena cava, acquisition of inflow and outflow control (without necessarily clamping the vessels) and parenchymal transection until the involved hepatic vein is met. A clamp is then placed at the origin of the hepatic vein and resection is accomplished with completion of parenchymal transection and sectioning all hepatic veins. When the hepatic veins are involved very close to the IVC, ante situm or ex situ procedures may be necessary, with or without HP [19,57,58,59]. In any case, the reconstructive phase necessitates TVE of the liver and HP (as described above), as well as porto-systemic or portocaval shunting, which can be considered depending on the time needed for reconstruction [36,58]. Reconstruction of the hepatic vein may include a patch, or anastomosis to the IVC or an IVC prosthesis [19,33,58]. Options for reconstruction include direct anastomosis, synthetic grafts, cryopreserved vein grafts or autologous grafts. Direct anastomosis is the ideal method when there is enough hepatic vein length to technically perform the anastomosis, but in most cases, a graft is needed. Much of the discussion on the different kind of grafts mirrors the considerations made for IVC grafts. Two further interesting options have been reported for hepatic vein reconstruction: jugular vein procuring or use of a PV graft harvested from the resected hemi-liver [19,59,60]. Although this surgical procedure may be complex and morbid, results are generally favourable, with a reported 12% mortality rate [19].

In some cases, alternatives to resection and reconstruction exist and can be pursued. For example, an inferior right hepatic vein (IRHV) can be present in up to 25% of livers [61]. In these patients, the IRHV may be enough to drain the liver, bypassing the necessity of right hepatic vein reconstruction/re-implantation after its resection with the involved IVC. Therefore, careful study of preoperative computer tomography can assist in the search for less morbid solutions. If flow through the IRHV is not convincing, embolisation of the proper right hepatic vein may allow increased transit and optimal postoperative results [62]. Other authors performed reconstruction of the middle hepatic vein when the FLR volume was <40% of total liver volume to decrease hepatic congestion and induce a volume increase [63].

#### 3.2.3. Central/Hilar Involvement

Surgical techniques used for iCCA involving hilar structures are the same as those used for pCCA (described in the following section).

## 4. Perihilar Cholangiocarcinoma with Vascular Involvement

### 4.1. Oncologic Considerations

Due to the tight anatomical relationships, vascular infiltration from pCCA usually indicates involvement of large portal branches or the main PV trunk and/or arterial involvement. Van Vugt et al. studied the prognostic significance of vascular involvement in 674 patients with pCCA [64]. Unilateral PV involvement did not affect median survival, while main PV or bilateral portal involvement was associated with reduced survival in a univariate, but not multivariate, analysis. Hepatic arterial involvement significantly reduced survival, whether unilateral or bilateral/main, and was confirmed to be an independent prognostic risk factor by multivariate analysis.

Extent and laterality of hepatectomy are mainly determined by neoplastic biliary extension. Contralateral vascular infiltration has been considered a contraindication for surgery for many years. This paradigm has changed in the last two decades, with many investigators reporting acceptable outcomes for vascular resection and reconstruction for pCCA. Nonetheless, current guidelines do not recommend routine vascular resection due to doubts that the benefits justify the significant surgical morbidity/mortality, and counsel that these operations should be undertaken only in the most experienced hepatobiliary centres [2,65].

Today, PV resection is performed with relative frequency in high-volume centres, where it is included in as many as 35% of operations for pCCA [66]. When PV resection is performed, mortality rates are approximately 3–5%, which does not differ from standard major liver resection, although rates as high as 12% have been reported [67,68,69,70]. A recent meta-analysis considering only very large volume institutions [71] reported similar mortality rates of resected pCCA with or without PV resection. PV resection permits the achievement of R0 resection in approximately 85% of patients. This is of relevance considering that R0 resection can double and triplicate survival, compared to R1 and R2, respectively [67]. In a multi-centre study by De Jong et al., long-term survival offered by PV resection was similar to those experienced by patients who underwent standard hepatectomy [67]. The results of main studies on PV resection are summarised in Table 3.

In a more recent single centre study, 303 of 787 patients undergoing surgical resection underwent vascular resection (either portal or arterial). The median OS was significantly shorter in the vascular resection group, compared to the standard resection group (30 vs. 61 months; *p* < 0.0001) [68]. Nonetheless, survival was significantly longer compared to patients who did not undergo resection (30 vs. 10 months; *p* < 0.001). Similar results were presented in a meta-analysis evaluating the outcomes of PV resection for pCCA [81]. In general, PV resection may offer long-term survival to these patients without additional morbidity/mortality. Based on this, Neuhaus et al. [75] advocated for routine “in principle” en bloc hilar resection with PV resection associated with left hepatectomy. This procedure was also named the “no-touch” technique and its long-term results are promising, since the one-, three- and five-year OS after hilar en bloc resection were 87%, 70% and 58%, respectively, which was significantly higher than after conventional major hepatectomy (*p* = 0.021) [75]. Unfortunately, these outstanding results were not replicated elsewhere [74,82].

Arterial resection for pCCA is still a source of debate, as the first reports that appeared in 2000 and initial results were extremely discouraging. In 2007, Miyazaki et al. [72] published their series of pCCA resections with and without vascular resection, including nine arterial resections. As the intra-operative mortality was 11% and in-hospital mortality was 33%, the authors concluded that arterial resection was not justified due to high morbidity and little (if any) benefit. In 2010, Nagino et al. published their series of 50 consecutive patients undergoing simultaneous PV and arterial resection, reporting a morbidity rate of 50% and mortality rate of 2%, a R0 rate of 66% and five-year survival of 30% [83]. A recent update included 146 arterial resections with a 4% mortality rate and a median OS of 34 months [68]. In this series, no difference in survival rate was detected between patients undergoing portal or arterial resection.

In 2018, another Japanese group [71] proposed a new approach to Bismuth type I/II pCCA. The latter, as it often invades the right hepatic artery, is usually resected with a right hepatectomy and caudate lobectomy. However, some of these patients’ FLR may have been too small and not sufficiently increased with portal vein embolisation (PVE). In these cases, the authors described the feasibility of a left hepatectomy plus caudate lobectomy with concomitant resection and reconstruction of the right hepatic artery. In this series, 12 patients who underwent left hepatectomy with caudate lobectomy and right arterial resection were compared to 24 patients who underwent right hepatectomy; morbidity and mortality were similar, no reconstruction-related events occurred and long-term outcomes were comparable [71]. Hepatic artery resection is not yet a standard option, but the Nagoya experience has proved its feasibility and its benefits, including long-term survival.

Although LT is not yet an established option for iCCA, this procedure is a codified strategy with satisfactory outcomes in select patients with pCCA [84]. In particular, patients with unresectable pCCA (either due to locally advanced tumour with extensive vascular and/or biliary invasion precluding complete resection or because of poor hepatic function reserve predisposing the patient to post-hepatectomy liver failure) undergo neoadjuvant chemoradiation followed by transplantation, with a 5-year disease-free survival of 65% (although drop-out rates are still high, around 50%) [7,84]. The transplant team should be prepared for arterial and venous jump grafts for hepatic artery and PV reconstructions [7]. In this context, PV encasement and perivascular invasion have been identified as risk factors for recurrence [85,86].

Involvement of the hepatic veins or IVC by pCCA is quite rare. When this does happen, it may be associated with concomitant hilar vascular invasion, forcing ex situ and autotransplantation techniques; however, the scarcity of reported cases does not permit evaluation of outcomes.

### 4.2. Surgical Approaches in pCCA with Vascular Involvement

#### 4.2.1. Inferior Vena Cava/Hepatic Vein Involvement

The surgical techniques used for pCCA involving the IVC or hepatic veins are the same used for iCCA (described in the preceding section).

#### 4.2.2. Portal Vein Involvement

As pCCA develops near the PV bifurcation, both the confluence and left (LPV) and right (RPV) branches can be involved in the disease process.

**Figure 4:**
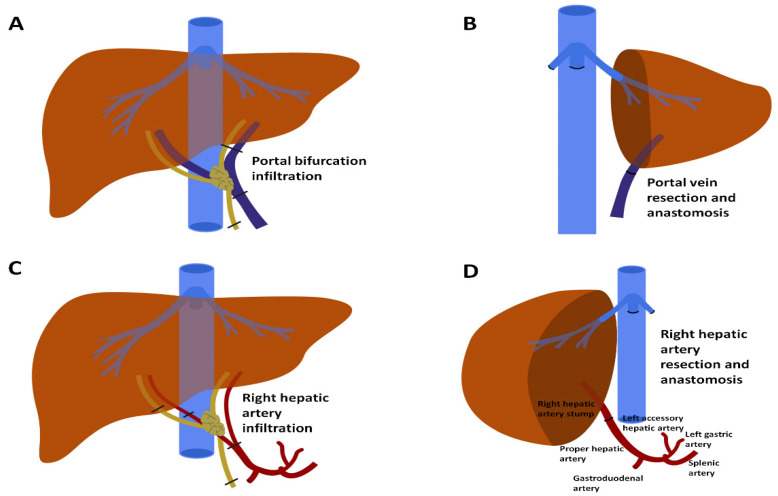
Resection and reconstruction for hilar cholangiocarcinoma involving portal vein or hepatic artery. (**A**) Hilar cholangiocarcinoma involving the portal bifurcation. (**B**) Resection has been performed, with portal end-to-end anastomosis. (**C**) Hilar cholangiocarcinoma involving the right hepatic artery. (**D**) Resection and reconstruction with end-to-end anastomosis between the right hepatic artery stump and the proper hepatic artery. Reconstruction is also possible using a rotating arterial graft with any of the named arteries in the picture. Finally, reconstruction with a radial artery graft or a saphenous vein graft is also possible (not depicted).

When the LPV is involved in a patient undergoing right hepatectomy, its resection and reconstruction are necessary. This reconstruction is usually straightforward as the LPV has a long extrahepatic course. Resection can be performed indifferently either before or after parenchymal transection. Most of the time, it is possible to reconstruct it by direct end-to-end anastomosis [70] (Figure 4A,B). Otherwise, grafts can be used (i.e., autologous iliac/jugular veins, cadaveric iliac veins, synthetic grafts).

In the case of RPV involvement, given its shorter length, reconstruction could be technically challenging. Direct anastomosis is rarely possible and if resection is conducted up to the first-order branches, reconstruction may then involve an autologous or cadaveric iliac Y-graft. During extended left hepatectomy, the main PV and right posterior branch could feature a large size discrepancy. For these reasons, wedge resections are more commonly attempted on the right side, followed by direct suture closure or patch closure (using cadaveric vein/peritoneum) [33,70,87]. Ebata et al. reported direct closure to be appropriate in most cases (90%) [70].

The “no-touch” technique or “a priori en bloc hilar resection” proposed by Neuhaus et al. entails right hepatectomy with systematic PV resection and reconstruction [75,88]. Hilar structures are dissected as little as possible: the right hepatic artery is divided just after its stemming from the proper hepatic artery, the main PV is sectioned away from the tumour and the LPV is divided just after its entering of the umbilical fissure. In this way, hilar dissection is largely avoided. Reconstruction is then performed, mostly with direct end-to-end anastomosis.

#### 4.2.3. Hepatic Artery Involvement

Hepatic arterial involvement in pCCA is much more common on the right side, since the right hepatic artery is normally located just beneath the common bile duct in the hilum, whereas the left hepatic artery usually travels at a distance. Arterial resection is usually performed last, just before excising the specimen. Nonetheless, it can be performed early, as long as the main principle is respected: immediate reconstruction, minimising liver remnant ischaemia time. De Santibañes et al. claimed pre-excisional arterial resection and reconstruction to provide the major advantage of giving the surgeon an opportunity to halt the operation before transection is begun, if satisfying reconstruction cannot be achieved [89]. End-to-end direct arterial anastomosis is the most common reconstruction method [68] (Figure 4C,D). In the largest single-centre series, end-to-end anastomosis was possible in 59% of cases [68]. In three of 89 cases (3.3%), end-to-end anastomosis “failed” intraoperatively due to thrombosis/insufficient blood flow and a secondary reconstruction method had to be used. Other reconstructive options entail the use of a rotating artery or an interpositional graft. Rotating grafts make use of arteries conveniently located nearby, possibly with the most adequate calibre. For this purpose, any of the following vessels are appropriate: gastroduodenal artery, left hepatic artery, left gastric artery, right gastric artery or splenic artery (Figure 4D). Alternatively, a jump graft between the proximal and distal cut ends of the hepatic artery can be fashioned. Interpositional grafts are commonly retrieved from the radial artery and greater saphenous vein. Comparative results of direct anastomosis vs. rotating artery or interposition grafts are not currently available.

Hu et al. describe a case series in which reconstruction was not attempted if ischaemic demarcation was not seen intraoperatively upon clamping/resection [90]. In this study, 29 patients with arterial resection without reconstruction were compared to 34 patients with arterial resection and reconstruction. There were no differences in major postoperative complications, mortality or long-term survival among the two groups. Peng et al. analysed results from 26 patients who underwent arterial resection without reconstruction and three who underwent standard left hepatectomy, and increased morbidity or mortality were not reported in patients undergoing arterial resection [91]. When this strategy is considered, right lobe mobilisation should be minimal to preserve collateral circulation from the diaphragmatic and retroperitoneal arteries. Furthermore, to enhance chances of success, Yasuda et al. described preoperative proper hepatic artery (or both sided) embolisation, increasing collateral flow, three weeks prior to left hepatectomy [92].

When arterial reconstruction is not deemed possible, and reconstruction is not an option (in cases of absent collateral circulation, absent Doppler-confirmed intrahepatic arterial flow or ischaemic change in colour), an artero-portal shunt (APS) could be performed as a salvage procedure. This procedure involves fashioning of an end-to-side anastomosis between the common hepatic artery and main PV [93], re-establishing sufficient hepatic oxygenation. The shunt can be radiologically obliterated later if collateral formation is demonstrated. Noji et al. investigated whether the APS procedure could serve a primary role as an alternative to hepatic arterial reconstruction [94], as the authors reported acceptable results of APS, but with significantly increased liver abscess formation compared to patients undergoing arterial anastomosis, and concluded that APS should be reserved as a salvage procedure when arterial anastomosis is not possible.

#### 4.2.4. Combined Portal and Arterial Involvement

When both the PV and artery are involved, resection and reconstruction of both can be undertaken. When both are resected simultaneously, the portal anastomosis is generally performed first [68]. When feasible, one vessel should be resected and reconstructed at a time while the other is patent, maintaining perfusion of the parenchyma and limiting ischaemic damage.

## 5. Conclusions

Radical surgical treatment remains the only curative option for CCA with vascular involvement. For CCA requiring complex vascular resection and reconstruction, the recent advantages of surgical techniques and satisfactory outcomes, in terms of complete tumour excision, justify the aggressive surgical approaches in selected patients. However, these surgical procedures might be extremely complex, yielding elevated risks, and continue to present fairly high mortality and morbidity rates. Therefore, careful evaluation by experienced hepatobiliary surgeons in a multidisciplinary setting is highly recommended in order to achieve favourable outcomes.

## Figures and Tables

**Table 1:** Major studies on IVC resection.

Study	Number of Patients	Major Morbidity (%)	Mortality (%)	Survival
Hemming [23]	60	NA	8.00%	35% 5 years OS
Malde [24]	35	NA	11.43%	Median OS 29 months
Nuzzo [15]	23	39.13%	4.35%	NA
Azoulay [17]	22	NA	4.55%	38.30% 5 years OS
Nardo [25]	19	NA	5.90%	Median OS 32 months
Sarmiento [26]	19	NA	5.26%	Median OS 38 months

Abbreviations: NA, not available; OS, overall survival.

**Table 2:** Major studies on in situ hypothermic perfusion technique (excluding case series with less than 5 cases or where technique-specific outcomes were not available).

Study	Number of Patients	Major Morbidity (%)	Mortality (%)	Survival (%)
Fortner [46]	29	NA	10.34%	59.09% at 30 months
Azoulay [13]	77	27.30%	19.48%	30.40% at 5 years
Navez [42]	27	40.10%	18.52%	NA

**Table 3:** Major studies on portal vein resection for pCCA.

Study	Number of Patients	Major Morbidity (%)	Mortality (%)	Survival
Ebata [70]	52	2.90%	9.60%	9.90% 5 years OS
Miyazaki [72]	34	38.00%	8.82%	16.00% 5 years OS
Song [73]	51	NA	9.80%	22.80% 5 years OS
Hemming [69]	42	NA	2.0%	NA
Tamoto [74]	36	58.33%	2.78%	Median OS 20.5 months
Neuhaus [75]	50	NA	NA	58.00% 5 years OS
Wang [76]	16	37.50%	0.00%	Median OS 20 months
Molina [77]	23	NA	22.00%	NA
Schimizzi [78]	31	47.37%	15.79%	Median OS 24 months
Mizuno [68]	157	48.00%	3.00%	Median OS 30 months
Kim [79]	35	34.30%	2.90%	37–70% 5 years = S
Kuriyama [80]	31	33.33%	3.40%	37.60% 5 years OS

## Data Availability

Data are contained within the article.
